# Social innovation in health and community-driven engagement as a key strategy for addressing COVID-19 crisis challenges: insights and reflections from the multicultural society of Iran

**DOI:** 10.3389/fpubh.2023.1174385

**Published:** 2023-06-06

**Authors:** Maryam Khazaee-Pool, Tahereh Pashaei, Koen Ponnet

**Affiliations:** ^1^Department of Health Education and Promotion, School of Health, Health Sciences Research Center, Mazandaran University of Medical Sciences, Sari, Iran; ^2^Social Determinants of Health Research Center, Research Institute for Health Development, Kurdistan University of Medical Sciences, Sanandaj, Iran; ^3^Department of Communication Sciences, imec-mict-Ghent University, Ghent, Belgium

**Keywords:** social innovation, community engagement, preventive strategies, COVID-19 crisis, crisis challenges, multicultural society of Iran

## Abstract

**Background:**

Social innovation is one of the strategies for appealing to people and encouraging social cooperation and engagement in interventions during crisis periods. In this regard, community engagement is an operative and innovative community health approach for achieving successful health outcomes. There is limited information about the role and operational impact of social innovation on community engagement during the challenges posed by the COVID-19 crisis. In this study, we aim to contribute to the understanding of innovative social strategies to attract social participation in crises such as the COVID-19 pandemic by highlighting the experience of social innovative strategies based on community-driven engagement in Iran.

**Methods:**

This qualitative study was conducted in seven provinces of Iran—Mazandaran, Zanjan, Golestan, Lorestan, Tehran, Kurdistan, and Khuzestan—from 4 September 2021 to 1 March 2022. A sample of Iranians (15–71 years) was selected by purposeful and snowball sampling methods to participate in the study, and 187 semi-structured telephone interviews were conducted. Participants were recruited from three levels of the community: community leaders, healthcare providers, and laypeople. The data collection tool was an interview guide, which was designed based on a review of the literature. The data were analyzed using conventional content analysis. Exploratory analyses were performed to identify social innovative strategies based on community engagement used during the COVID-19 crisis in Iran. The interviews continued until data saturation was reached.

**Results:**

Based on our findings, we distilled innovative strategies into 6 main themes and 37 categories: (1) information giving/sharing, (2) consultation, (3) involvement/collaboration, (4) health education and prevention, (5) empowering, and (6) advocacy. The results revealed that the participants were very driven to engage in the management and control of the COVID-19 crisis, even though they faced significant challenges.

**Conclusion:**

The spread of the COVID-19 pandemic required social- and community-based responses. These reactions increased the possibility of fair access to health services, especially for vulnerable groups and minorities. As with other epidemics, applying the experience of the comprehensive participation of communities played an important and active role in the prevention and control of COVID-19. In this regard, giving and sharing information, consultation, involvement/collaboration, health education/prevention, empowerment, and advocacy are the most important innovative strategies that might encourage the community to perform COVID-19 crisis management and control.

## Introduction

The COVID-19 pandemic was sudden and shocking, resulting in severe changes that affected communities. The crisis was not a singular phenomenon but was influenced by sociocultural, political, and economic components of community settings, resulting in several negative impacts that could not be adequately handled by current and routine strategies. Therefore, the most effective global strategy was to use indigenous and native approaches that fit the sociocultural context to reduce the spread of infectious diseases. Approaches such as individual and community empowerment become relevant within cultural contexts to identify cultural factors that affect health and to remove obstacles and challenges related to health. Social innovations for health interventions can meet these challenges (1).

Social innovations are social goals linked to the successful lives of society to reach satisfaction through a well-organized, active, reasonable, and practical response to current needs (2). Social innovations are novelties that are communal, both in their approaches and in their objectives, and they target the detection of novel reactions to social complications and challenges by recognizing and offering novel facilities that grow the lives of persons in the community (3). The relationship between innovation and society is a complex issue (4). When culturally consistent solutions are combined with responsive care processes and existing local governance structures, opportunities for improving health and social innovation are created (5). Typically, reactions to public health crises include top-down government activities and community health organizations. This usually involves holding discussions with the community and using biosecurity methods, as well as considering their cultural diversity (2, 6).

Multicultural societies regularly display the necessity of culturally stable strategies for individuals during a health crisis and in confronting unequal health problems (7, 8). When such culturally stable strategies are merged with accountable care procedures and current local governance constructions, chances of progressing social innovation and health are generated (6). When encountering a health crisis or pandemic, societies noticeably request and look for descriptions as a reaction to their vulnerabilities and being in danger (7). Diverse cultural systems are expected to have varying disease perceptions during a pandemic, leading to differing coping approaches (9). This becomes even more crucial in multicultural and multireligious contexts, such as Iran (10). Iran is an interesting example of a country with great diversity in ethnic, linguistic, religious, and cultural groups, including Persians, Kurds, Lurs, Mazandaranis, Gilakis, Azerbaijanis, Arabs, Balochi, and Turkmens. The nation is also home to several minority groups, including tribal Turkic groups, Talysh, Armenians, Georgians, Assyrians, Jews, Circassians, Russians, Koreans, and Iraqis (11). The differences and cultural diversity in Iran can pose different behavioral reactions during health-related crises in the community.

It is important for middle-income or low-income communities, especially those with diverse cultural and ethnic characteristics, to recognize how they will be influenced by a public health crisis and how to distinguish their barriers and challenges. One of the practical and useful public health approaches for strengthening social innovation in health in poor communities, growing health consequences, and certifying social support is to promote community engagement (CE) (3). CE is defined as “a procedure of rising interactions that allow a community to confront each member to consider health-related topics and increase health to attain good health effects and results.” The concept of CE refers to fostering conditions and occasions for community members to have their opinions acknowledged in identifying the challenges and suggesting strategies to resolve the health difficulties they face (3). The risk of contracting the COVID-19 crisis cannot be entirely attributed to individual threats, as it also involves wider sociocultural and structural factors of health that lead to inequities in communities where at-risk people live, act, perform, and acquire (12).

For effective CE during a pandemic, such as COVID-19, there must be an environment with a common culture and purpose and sufficient levels of cross-cultural abilities in controlling the crisis, which may lead to the more effective implementation of pandemic strategies and possibly reduce the inequity that may be presented throughout the pandemic (13–18). In particular, the unequal burden of COVID-19 death was pronounced among multicultural populations in societies that have a history of providing unequal health services (13–16). Therefore, CE is vital for disclosing the opinions of those in the community using culturally appropriate strategies that are more likely to remain stable beyond the COVID-19 crisis. To perceive these communities, the role of culture is important if any strategy is to be accepted or stabilized (8). Culture is key to effectively informing the community about COVID-19 for CE, and it is also important for its universal response to CE (19). The community includes community leaders, community networks or teams, health-controlling groups, persons, and main stakeholders (elderly, schoolchildren, youth, females, and others susceptible) (17, 20). In response to crises, an approach that entails full community participation is recommended, including the broad ability of all areas, such as trade, non-profit organizations (NGOs), groups, and the public (2, 17).

Previous studies have revealed the positive impacts of social innovations on the community-based distribution of health facilities and the mediating role of native strategies for infection prevention/control and CE. The positive effect of engaging communities to address neglected tropical diseases or planning for an influenza pandemic among disadvantaged groups has been reported (13–18, 20–23). In low- and middle-income states, CE has been a vital activator of dynamic reactions to direct communicable infections (24). CE was operative in responding to the 2014 Ebola epidemic in the setting of a poor health organization in Sierra Leone, where civic answer groups helped curb the native spread of the disease via contact finding, house-to-house appointments, health services, and community reporting (25, 26). Furthermore, CE promoted a significant reduction in children's deaths due to malaria in Ethiopia, as well as a decrease in HIV incidence among the people (24, 27).

Even when structures are present for engaging community participants in social innovations, the particular approaches implemented must reveal and be accepted in the native background, values, and legislation (21). The newness of COVID-19 has offered exclusive challenges that current models of facility supply may not be sufficiently designed to tackle (28). Therefore, it is essential to develop socially innovative strategies and approaches based on CEs to confront the challenges arising from the COVID-19 crisis (29). Thus, guided by the literature on social innovations (30) and CE (17, 31), this study explores how social innovations in health based on community-driven engagement can be leveraged to react to the health requirements, barriers, and challenges resulting from COVID-19 in the multicultural society of Iran. Until now, there has been little understanding of social innovation practical strategies for CE in confirming the adoption of government strategies for monitoring COVID-19 in multicultural societies, such as Iran. In the current project, we aim to fill this gap by highlighting the experience of CE during the COVID-19 crisis in Iran to provide insights that may enhance knowledge of social innovations through CE in multicultural societies.

## Methods

The purpose of our qualitative study is to identify social innovative approaches that might increase community participation in response to the COVID-19 crisis.

### Study design and setting

This was a qualitative content analysis study conducted from 4 September 2021 to 1 March 2022. We employed conventional content analysis, given the limited data in the field of study at that time of the study in the Iranian context; thus, there were no preconceived hypotheses. Semi-structured individual interviews were used to gather data in the study. Each interview continued for not more than 40 min to avoid the mental fatigue and weariness of the interviewees. This study was conducted in seven provinces of Iran, including Mazandaran, Zanjan, Golestan, Lorestan, Tehran, Kurdistan, and Khuzestan, reflecting Iranian society's diverse cultural and linguistic features. Each of these provinces has a different language, accent, and cultural characteristics.

### Participants

Participants were recruited from three levels of the community: community leaders, healthcare providers, and laypeople. Community leaders included community chair people, town, quarter, mayors/village headmen, school/university superintendents, religious leaders, business leaders, union leaders, imams, and other well-known community figures. Participants were also selected at the national level, including senior-level policymakers from two government offices (*n* = 5) and seven NGOs (*n* = 9).

To obtain different perspectives, participants were chosen by both purposeful (with maximum variation) and snowball sampling due to the necessity of sample diversity in terms of demographic characteristics and expertise (32). However, most participants in this study were recruited by purposeful sampling. In the selection of participants, the maximum variation or “heterogeneous method” was used to achieve varying levels of socioeconomic status, ethnic, linguistic, demographic, age, gender, level of education, and place of residence. Given the diversity of the culture and ethnicity of Iranian society, we tried to invite most of the ethnicities that covered both urban and rural communities for interviews. Snowball sampling was used on a case basis by encouraging recruited participants to invite others to participate in the study. At the launch of the study, the purpose of the study was explained to each participant. Interviewing with participants was continued until data saturation was achieved; that is, no new codes were discovered in the data.

### Data collection

The data gathering technique in this research was telephone-based semi-structured individual interviews. At the start of each interview, the participants were asked about their demographic characteristics. An interview guide was used for the interviews, which was designed based on the literature review and the views of several professors in the fields related to the topic and research. The interviewing process is described in detail in Additional File 1.

Considering the dispersion of the study setting, the cultural and linguistic diversity of the research community, the high prevalence of COVID-19, and the time efficiency of the study, four researchers familiar with qualitative research methods were invited for administrative coordination to obtain the participants' contact numbers. In this regard, for the provinces of Khuzestan, Zanjan, Lorestan, and Golestan, which have various languages and dialects, native researchers who were also familiar with qualitative research methods were invited to participate in the study. The first author of this article conducted interviews in Mazandaran and Tehran provinces, and the second author of this article conducted interviews in Kurdistan province due to familiarity with the local language of that region. The reason for this choice was that these people were familiar with the culture, language, and religious characteristics of the mentioned areas. After the administrative coordination of the different departments by each researcher in the selected provinces, the contact numbers of the individuals were collected to obtain permission and coordinate the time of the interviews.

In total, seven trained interviewers who were familiar with the culture of the selected provinces as well as qualitative research methods conducted semi-structured 40-min telephone interviews with 187 participants. Data collection lasted from September 2021 to March 2022. The interviews were conducted on Android smartphones that had the call-recording property. Telephone interviews were conducted due to the physical distancing protocol and the necessity of maintaining safety while conducting the qualitative study. Each interview continued for not more than 40 min to avoid the interview fatigue of the interviewees. Most of the interviews were held in Farsi (the official language of Iran). In cases where the participant preferred to speak his native (mother tongue) language or could not speak Farsi fluently, the interview was conducted in the native language of the same region (which was the reason for selecting interviewers familiar with the language of the selected provinces).

The participants were encouraged to discuss their experiences with innovative approaches that increased CE to control the COVID-19 crisis. Similarly, they discussed sociocultural and ecological components that might have had an effect on the level of CE regarding this crisis. The interviews focused on the following three main questions:

- How was your experience with engagement in the management and control of the COVID-19 pandemic?
- What strategy and procedure have you applied for engagement in the management COVID-19 pandemic?
- How have the strategies and procedures affected your engagement in this regard?

Based on responses to the questions, follow-up questions were asked. After each question, participants were invited to explain more about what they had reported. For example, they were asked, “What do you mean?” or “Explain more” for a deeper consideration of the participant's experiences regarding the topic.

To maintain the confidentiality of the participants' information, the interviews were numbered from 1 to 187. After 183 interviews, the data were saturated. The interviews were recorded and transcribed verbatim in Farsi. Considering that some of the interviews were conducted in the native language of the people of that area, after the interviews, the recordings were translated into Persian by the same interviewer. The transcripts and digital recordings were cross-checked.

### Data analysis

Content analysis with a conventional approach was used to explore the information based on Graneheim and Landman's approach, identify main themes, and compare patterns through several individuals (33). All interviews were audio-recorded after obtaining informed consent, and the audio-recordings were listened to carefully several times. The recordings were transcribed word by word into Persian. The researchers then read the interviews numerous times and discussed the best coding method. All recorded transcripts were converted into meaning units. Concepts of key words and expressions concerning the interviews' context were built. Concepts were displayed as codes after the completion of all interviews. The primary code was obtained, the meaning unit was summarized into a “condensed meaning unit,” and the extracted codes were created in the next step. After the coding structure was identified, the interviewer entered the transcripts into MAXQDA software v12, which allowed the text to be coded and restored for ease of interpretation. The study group compared all transcript stages to categorize code relationships and differences. The researchers then explored the differences and reconciled them, and the final code was extracted. Similar codes formed subcategories, and the main categories were created from subcategories. Finally, the themes were obtained based on some related main categories. All audio files and transcripts are accessible to reviewers.

### Rigor

The rigor of the data collection was confirmed by analyzing its credibility, transferability, confirmability, and dependability (34). In the current study, several features of trustworthiness were identified. Credibility was established through lasting engagement with information, member checking, and peer debriefing. We invited a second coder who was trained in qualitative study. We requested seven of our participants to review the transcripts, a summary of the interviews, and the developing results (member check); these participants were selected across the interviews. Confirmability of this study was achieved by sending content codes and themes to six researchers familiar with qualitative content analysis methods (peer checks). The transferability of the current research was ensured by providing a rich and complete explanation and a detailed report of the study method. The transferability of this study was ascertained by using the maximum variation sampling method (35). The dependability and credibility of the present data were confirmed by the obvious coding method and inter-coder confirmation (34). Additionally, data were analyzed using a thematic analysis method in Farsi, and the codes and study information were transcribed in English.

### Ethical considerations

The study received ethics approval from the Mazandaran University of Medical Sciences Review Committee (Approval No/2021.06.23/IR.MAZUMS.REC.1400.250; Grant No. 11483). All participants were specified using aliases and were informed that their contribution to the current study was voluntary, they could withdraw at any time, their privacy would be maintained, and none of them would be recognizable in any publications resulting from the study. Informed consent was collected from all participants before the interviews.

## Results

In total, 187 Iranian people aged 15–71 years (41.5 ± 8.71 years) took part in the study. The characteristics of the participants are reported in Table 1. Overall, six major themes emerged from the analysis: (1) information giving and sharing, (2) consultation, (3) involvement and collaboration, (4) health education and prevention, (5) empowering, and (6) advocacy. More information on the themes and categories is presented in Table 2. However, in the following section, we reflect on the participants' experiences of major themes.

**Table 1:** Socio-demographic characteristics of participants (*n* = 187).

**Characteristics**	**Values**, ***n*** **(%)**	
Age (years)		
	15–34	69 (36.9)
	35–54	93 (49.7)
	≥55	25 (13.4)
Gender		
	Male	85 (45.5)
	Female	102 (54.5)
Education		
	Primary	37 (19.8)
	Secondary	45 (24.1)
	Higher	105 (56.1)
Occupation		
	Government's employee	67 (35.8)
	Freelance job	55 (29.4)
	Retired	14 (7.5)
	Unemployed	51 (27.3)
Ethnicity		
	Persians	43 (23)
	Mazanderanis	29 (15.5)
	Gilaks	17 (9.1)
	Kurds	19 (10.2)
	Lurs	12 (6.4)
	Azerbaijanis	18 (9.6)
	Talysh/Tats	9 (4.8)
	Baloch	14 (7.5)
	Turkmen	9 (4.8)
	Arabs	17 (9.1)
Current region of residence		
	North	44 (23.5)
	South	32 (17.2)
	East	37 (19.8)
	West	33 (17.6)
	Center	41 (21.9)

**Table 2:** Main themes and categories.

**Main themes**	**Categories**
Information giving/sharing	
	Public awareness and health literacy campaigns
	Correct information management
	New technologies and telecommunication tools
	Fliers and local advertising
	Media infrastructure
Consultation	
	Getting feedback from community
	Consultation with community leaders/stakeholders
	Setting up public and open consultation systems
	Launching a self-assessment system
	Comprehensive integrated system for psychosocial health services
Involvement and collaboration	
	Networking
	Lobbying between government and NGOs
	Multi-sectoral collaboration through COVID-19 committees
	Establishing platforms
	Social mobilization
	Shared leadership/decentralization/ability to control
	Managers' commitment
	Incentives and motivations
	Promoting a culture of participation
	Sensitizing, persuasion, and pressuring
	Stakeholder engagement
Health education and prevention	
	Prevention
	Risk communication
	Screening
	Quarantine
	Maintenance
Empowering	
	Jihadi, voluntary, and faith-driven actions
	Social trust building
	Social responsibility
	Community resiliency
	Strengthening society efficiency/finding talent
Advocacy	
	Leaders involvement (legislators; policy makers; decision makers)
	Partnership building and coalition
	Mass/virtual media and digital health
	Mobilizing the community groups
	Capacity building
	Legal and policy-making strategies

### Theme 1: Information giving and sharing

One of the themes produced in the current study was information giving/sharing. Here, information giving/sharing describes the exchange of data about the COVID-19 pandemic between various people and organizations (public, private, and non-governmental institutions and organizations) in different ways, such as the use of technologies. Based on the participants' comments, the five key strategies for sharing information related to the recent crisis were: (1) public awareness campaigns, (2) information management, (3) new technologies and telecommunication tools, (4) local advertising (with billboard/banners for training and TV messaging), and (5) media infrastructure (mass media and cyberspace).

During the COVID-19 crisis, information giving/sharing was used to raise information awareness about the COVID-19 disease, building awareness and sensitivity to accept health protocols, increasing perceived sensitivity, and advertising via social media. At the beginning of the COVID-19 crisis, rumors and misinformation were circulating via different communication channels. Therefore, one effective strategy during this crisis was accurate information management. This included accepting the worries of the community, offering occasions for conversation, and countering rumors, false information, and infodemic by social media, national/local media, and infographics.

“*In line with education and information in the field of* COVID-19 *disease and vaccination and the importance of awareness in this regard, all educational clips related to* COVID-19 *are available on Avay Salamat website at https://iec.behdasht.gov.ir (in the health campaigns section/Let's stay together). The site is loaded …”* (Participant 14)

Participants reported that insufficient and sometimes inconsistent information provided by the government and health workers about COVID-19 caused them to feel anxious and distrustful.

“*The government was giving citizens one kind of information about COVID-19, and maybe private organizations, media, and even NGOs were giving another type of information and content about the way COVID-19 virus is transmitted. So, at the beginning of the COVID-19 epidemic, everything was mystifying and even contradictory.”* (Participant 56)

Another strategy used by the government and NGOs to inform the community was technology, especially in awareness campaigns about COVID-19. For instance, to inform people, the Association for Support of Children with Cancer (Taskin) carried out a campaign in the suburbs of Tabriz, with the participation of volunteers and the Tabriz Municipality. The educational campaign of Halal Houses titled “Stay Together” was held with the participation of the International Committee of the Red Cross and the Health Education/Promotion Office of the Iranian Ministry of Health from December to March 2021. Other educational campaigns were “I Will Not Touch You,” “Stay At Home,” “Aware Society,” “Neither to Sanctions Nor to Corona,” “Let's Stay Together,” etc. Furthermore, the director of public relations at a university of medical sciences said:

“*We launched the “Ham-Ghasm” campaign with the aim of increasing community awareness and emphasizing the role of citizens in the management of Corona, so that we encouraged people to participate in controlling the disease … We tried to inform our fellow citizens that they should not leave their homes except in emergency moments …” (Participant 91)*

The communities were also informed through social media and virtual space, including smart SMS notifications and alerts (sending text messages to infected people who had violated quarantine and high-risk people). The educational messages about the coronavirus compiled by the Ministry of Health included information such as staying at home, wearing a mask, washing hands, social distancing, vaccination against COVID-19, and a respiratory mask educational guide.

### Theme 2: Consultation

In this study, the purpose of consultation (based on the participants' viewpoints) included all the effective strategies used by the government and health system employees to increase interaction with the community, attract community participation, and reduce psychological pressure caused by the spread of the COVID-19 pandemic. This theme encompassed five sub-themes: (1) getting feedback from the community, (2) consultation with community leaders/stakeholders, (3) setting up open consultation systems, (4) launching a self-assessment system, and (5) a comprehensive integrated system for psychosocial health services in the health network.

At the beginning of the COVID-19 pandemic, the use of counseling was not very active, although it was indicated as an effective and complementary approach by the government to reduce the psychological pressure caused by the disease, to reduce the socio-economic problems caused by it, and to increase community participation. In this regard, one of the participants noted:

“*I totally remember. At the beginning of Corona, my family was terrified. Everything was closed, and even counseling clinics were not safe [referring to the rapid spread of disease in gatherings and offices] … I had no faith in remote medicine and online counseling before this crisis … It was a novel and different [online consultation] for me … and we couldn't receive feedback physically at the clinic. So, I got the first consultation on Sky Room. This doctor recognized who I was, so we weren't completely unfamiliar with each other. Although it was a good experience for the first time.”* (Participant 64)

This form of CE occurred in some provinces of Iran during the COVID-19 crisis. One of the managers of the city health center defined their approach to attaining community reaction: “*In cooperation with non-governmental organizations and religious leaders, we formed the community engagement forum so that we could have a good interaction with the people of the region … We used the local media to invite people.”* (Participant 11)

The other strategies were setting up the counseling line 1,480 and social emergency 123, setting up an internet phone line center (4,030) with 2,000 lines and the possibility of contacting families and following them up, and having comprehensive telephone counseling services for children/teenagers (e.g., mental health services and psycho-social support). The expert of the welfare organization said:

“*The number of calls to the voice of the welfare consultant at number, 480 has increased more than 50% compared to previous years, which is due to the spread of Corona and people's preference for free and remote counseling in this situation. The welfare counseling hotline is active in the fields of family counseling, mental disorders, depression, anxiety, and child problems, and clients raise their problems in this system.” (Participant 81)*

One of the effective strategies during the COVID-19 crisis was setting up a self-assessment system for psychological disorders (intelligent screening) for the community and the prevention of psychosocial harm caused by this crisis, which was run by the welfare organization. The general director of the welfare of one of the provinces said:

“*In this situation, society needs psychological interventions, social health, and preventive interventions more than ever, and in this regard, the smart self-assessment system (smart screening) of the country's welfare organization has been launched at http://corona.behzisti.ir.”* (Participant 17)

Phone hotlines were introduced as a possible means for people to record worries and receive a consultation, although the hotlines were occasionally disconnected and consequently provoked more frustration than benefits. Other practical measures included the launch of an integrated comprehensive system of psychological health and community facilities throughout the outbreak of COVID-19 for providing remote psychosocial consultation services and holding psychotherapy.

### Theme 3: Involvement and collaboration

Collaboration refers to the commitment, cooperation, partnership, and participation of the community with the government in the management of COVID-19. Collaboration and involvement include improving the effectiveness of government through encouraging partnerships and cooperation within the government, across levels of government, and between the government and private institutions, as well as at the community level. It included all the ways and strategies that were synchronous or asynchronous, virtual, or in-person to increase the commitment to manage the COVID-19 crisis by the government and NGOs that were used. According to the opinion of the participants, 11 approaches of involvement and collaboration were used to increase community participation in the management of COVID-19: (1) networking; (2) lobbying between government/NGOs; (3) multi-sectoral collaboration through COVID-19 committees; (4) establishing platforms for community collaboration; (5) social mobilization; (6) shared leadership/decentralization/ability to control; (7) managers' commitment; (8) incentives and motivations; (9) promoting a culture of participation; (10) sensitizing, persuasion, and pressuring; and (11) and stakeholder engagement.

Crises similar to the COVID-19 epidemic focus on the worth of partnerships between government and community for durable crisis management achievement. Mainly in a disaster, governments must appeal to specialists with distinctive, cross-functional viewpoints to explain quickly varying, multipart complications that have long-term effects. The majority of participants agreed that crises such as the COVID-19 pandemic could be controlled by involvement and collaboration between community and government, including networking, collaboration, and lobbying between government and NGOs in a crisis, multi-sectoral collaboration through COVID-19 committees, establishing platforms for community collaboration, social mobilization and participation, using existing networks, managers' commitment, incentives, and motivations, promoting a culture of participation, health education and prevention, sensitizing, persuasion, and pressuring. One of the participants mentioned:

“*Community collaboration and involvement are when the public, patients, carers, amenity users, and other members of the public work in partnership with crisis management groups and apply their previous experience to participate in the plan, study, management, or dissemination of lessons learned.”* (Participant 94)

One of the key approaches to COVID-19 crisis management was women's willingness to collaborate in networks. One of the women said:

“*Women have always been able to play multiple social roles. A group of women, regardless of their age and education, have started to form local networks by using the capacities of civil society and in preparing hot homemade food, masks, or disinfecting public places.”* (Participant 82)

Participants were amazed at how respected their pre-existing unofficial networks (e.g., NGOs, local networks, social media, etc.) were in this way. They mentioned some effective elements in this regard, including knowledge networking, support management, dialogic loop of citizens on social media, customary/spiritual leaders, doctors, actors, and the college workforce.

“*We mostly talked to each other via a phone messaging or video platform [WhatsApp]. We used the messaging platform in COVID-19 to exchange food and domestic substances during the lockdown, in which limited shops in our village locked down, and access to vital items became hard.”* (Participant 88)

Most participants also emphasized cooperation with government organizations and native groups to develop effective collaboration through networks. One of the participants reported:

“*We tried to tap into present networks, including traditional leaders, doctors, heads of councils, spiritual leaders, actors, teachers, university staff, sports players, and actors.”* (Participant 23)

Other effective measures included collaborating and lobbying between the government and NGOs, charities, media, and labor unions to attract participation in this crisis management. A participant said:

“*I believed that a major part of this stable situation in different provinces is due to the cooperation of NGOs and popular and jihadi groups in persuading community and faithful help.”* (Participant 37)

Social mobilization, another strategy applied during the COVID-19 pandemic in Iran, was performed through the following activities: the cultural vow of masks, sending food and money to families whose businesses were closed, sending wedding/death expenses to the needy, and providing tablets and phones for underprivileged students. Designing sports and cooking clips/entertainment for families, launching innovative festivals, and designing applications were the effective measures taken by public and private organizations since the beginning of COVID-19 in Iran. Participants also recommended that the matching strengthening approaches of CE and trust building were important in managing the COVID-19 pandemic. According to one of the key informants' views:

“*… The battle against COVID-19 was fought because of people's efforts and community mobilization. Although the government provided various strategies, the eradication of COVID-19 is achieved by mobilizing the community … If I don't desire to arise, who come? … We have a professional commitment and responsibility.”* (Participant 44)

Promoting a culture of participation was another strategy for solving difficulties in cooperation and involvement with communities, such as official cooperation.

“*When the COVID-19 peaked, we held meetings via the village leaders. To prevent more spread of COVID-19 in the village, we assigned roles to people because village' people were familiar with the access points and recreation areas of the village. We bought handwashing equipment and masks with the financial support of the village council and benefactors and placed them at the entrance of the village and public places, including the bakery and 3 supermarkets of the village. Likewise, we put banners at the entrance of the village, stating that travel is prohibited.”* (Participant 50)

### Theme 4: Health education and prevention

The strategy of health education and health promotion in this study emphasizes all the individual, group, institutional, community, and systemic approaches that were used by the government, organizations, and society to improve the knowledge, attitudes, and behaviors in COVID-19 prevention. This strategy was tailored to the target population and community. This strategy aims to reduce health inequalities and discrimination so that all people in the COVID-19 era can fulfill their greatest health potential. This theme included five sub-themes: (1) prevention, (2) risk communication, (3) screening, (4) quarantine, and (5) maintenance.

Health communication emphasizes activities that can reduce the threat of COVID-19, such as how and what time to use a face mask, wash hands, and link to others, even though maintaining a suitable physical distance. In the offices, the body temperature of the employees was measured upon entering the building; masks were used, hands were disinfected, and a physical distance of 1–2 meters from other persons was maintained. One of the participants said:

“*If someone has come from COVID-19 pandemic state, or having some COVID-19 signs, their boss demanded that they stay/go home, or go to clinic or healthcare center for checkup. The bosses also requested individuals not go to the COVID-19 pandemic area.” (*Participant 18)

The COVID-19 screening included explaining the time and place the community could be screened, providing information about the costs of screening tests for COVID-19, and informing about safe activities. According to one of the participants' views:

“*We attempted to know the procedures published by the National Headquarters of Administrating COVID-19 and the Ministry of Health in Iran [regarding types of diagnostic screening tests for COVID-19]. We discussed with other friends and relatives their experiences in performing screening tests with COVID-19 and then transferred this awareness and experience to family.”* (Participant 37)

During the quarantine phase, participants obeyed the rules and guidelines on the management and control of the COVID-19 crisis. They used masks in public spaces. Some university students did not return to their birthplace because of the distress of spreading to their relatives. Another participant reported that:

“*I tried to track the new and updated quarantine guidelines during the COVID-19 era, and when the government announced a general quarantine, I only went out to buy essential items such as food and medicine. I was in the dormitory for 3–4 weeks and did not go to my hometown.”* (Participant 26)

### Theme 5: Empowering

Community empowerment is a key concept and strategy that discusses the process of enabling communities to increase control over their lives during the COVID-19 pandemic. In this study, community empowerment addresses Iran's sociocultural, political, and economic elements, as well as the infrastructure health of the Iranian community, and includes building partnerships with other sections in building community-based preventive strategies during the COVID-19 pandemic. This theme is divided into five sub-themes: (1) Jihadi, voluntary, and faith-driven actions; (2) social trust building; (3) social responsibility; (4) community resiliency; and (5) strengthening society's efficiency/finding talent.

Social organizations can respond to the COVID-19 crisis through appropriate practices and innovative strategies. One of these cases involved the voluntary sector, such as NGOs, which played a key role in empowering individuals/communities and attracting their participation through various social and economic support. One of the members of the board of the parliament's industries and mines committee said:

“*Identifying day laborers and women heads of families and helping them through support organizations such as Imam Khomeini (RA) Relief Committee is one of the necessities in the current situation. This is because, before the outbreak of Corona, due to the high rate of inflation, these people had financial problems. Before the spread of COVID-19, they faced many financial problems due to the high rate of inflation. In the current situation, they are dealing with much more difficult conditions due to the closure of daily wage businesses.”* (Participant 92)

Some of the jihadist and voluntary actions carried out to empower the community in the management of COVID-19 were performing different plans with the participation of local institutions (such as mosques, charity centers, NGOs, and health ambassadors), implementing culture-based plans (e.g., “Every Home Is a Health Base,” “Jihadi camps,” “Mosque-centered project,” and “Health Improvement Plan for Women and Girls [prisoners]), free visit, subsistence package, nursing home care, etc. One of the key informants stated:

“*The “Every Home is a Health Base” plan has been held with the main aim of appealing community and NGO involvement in attempts to manage the COVID-19 crisis. This plan is a perfect sample of fetching community engagement, interdepartmental coordination, organizing based on the desires of neighborhoods, and the best use of the latent of the nation's healthcare system…”* (Participant 87)

The participants stated that social trust building by the government plays an effective role in increasing CE. Examples of these approaches include having face-to-face interactions, cooperating with organizations trusted by society, and seeking support from neighborhood trustees. Some key informants stated that prior face-to-face communications were key to trust building, and some success they had in CE in the COVID-19 crisis reaction was due to previous efforts they had executed with them. Organizations resorted mostly to collaborating with communities if infrastructures such as the internet were accessible. One of the managers said:

“*Trust was vital for community participation. We invited famous people such as celebrities, athletes, artists, scientific, cultural, religious, and social figures to city conferences.”* (Participant 11)

Another prominent point raised by many participants in increasing community empowerment and, as a result, increasing CE was social responsibility. One of the participants stated:

“*In any case, all people are responsible for this crisis. It is not only the duty of the government … the government must guide and manage, and the people must participate. I used my personal van to disinfect the village's alleys at the start of the quarantine [during the Nowruz].”* (Participant 39)

The COVID-19 pandemic has created many challenges for all societies. However, it had more negative effects on vulnerable people (e.g., women, children, aging, and patients) than other communities. Among these negative effects, reference elements included business closures, women's unemployment, domestic violence, and the price of essential items. Therefore, according to the suggestion of most of the participants, one of the effective strategies for empowering them is to increase the community's resilience. An imam of a local mosque explained:

“*All the people of our village [the adolescents, the teachers, farmers, and village head], we together have collected money and dispersed lentils, rice, vegetables, detergent, etc. to the poor people. This is our social responsibility. Even the local baker covered some poor families in the village to receive as much free bread every day as they needed. Although these contributions are limited, they help to increase the resilience of poor families …”* (Participant 37).

### Theme 6: Advocacy

Advocacy has been used as one of the key strategies to promote community health during the COVID-19 pandemic. Advocacy for health as a combination of individual and social activities planned to achieve political commitment, social approval, policy support, and systems support for managing the COVID-19 crisis via CE. It takes into account actions and publications that affect public policy, public opinion, and laws. This theme consisted of six sub-themes: (1) leader involvement (legislators; policymakers; and decision makers), (2) partnership building and coalition, (3) mass/virtual media and digital health, (4) mobilizing community groups, (5) capacity building, and (6) legal and policymaking strategies.

In considering the role of communication during the COVID-19 crisis, advocacy is regularly mentioned as a key and important strategy of risk/health communication overall. This was a novel time when planning interventions with communities, patients, and other main stakeholders had never been key in expressing essential priorities, and community and patient desires. As the specific influences of the COVID-19 crisis were not similarly perceived, strategies must be community-detailed and addressed to the maximum key priorities. Health managers achieved support for plans by recognizing and involving local community leaders—religious leaders, head of the village council, head of the village, teachers, chiefs, elders, imams, vicars, and clerics—and consulting and cooperating with them as gatekeepers for access in a community. An NGO agent also restated the significance of employing local resources and communication techniques acquainted with the community:

“*Although the new technologies were effective, they were not the answer alone … What was very effective was the cooperation of the local leaders … In our village, the village council members installed a loudspeaker on an agricultural tractor, and then the chairman of the council took necessary measures to inform the public about preventing the spread of the disease.”* (Participant 73)

Combining informal settings (e.g., public gatherings, festivals, sports events, indoors) and formal settings (meetings, seminars, and conferences) helped the government attract community participation. A member of a city council said:

“*Our city council, in cooperation with the welfare department, launched a campaign of creative ideas as well as a children's painting festival about coronavirus and ways of managing it.”* (Participant 66)

Another practical advocacy strategy was partnership building and coalition. Most of the participants emphasized the key role of national and international NGOs, media, universities, public participation houses, and the clergy. A member of the NGO said:

“*Face-to-face interactions are very important for the promotion of non-governmental organizations, but many of them were lost during this era. However, many virtual links and media campaigns were launched during this period with less cost and no need to travel. Coalition building is the key to success in non-governmental organizations. Today, we can easily communicate with many people from all over the world and involve people in health campaigns.”* (Participant 62)

Mobilizing community groups was another advocacy strategy for CE during the COVID-19 crisis. Participants listed some different methods in this regard, such as engaging community leaders, using trusted religious figures in neighborhoods to encourage people to inject vaccines, and involving key groups (athletes, actors, religious leaders, and neighborhood councils). Capacity building was another advocacy approach emphasized by interviewees. The participants mentioned several examples of community capacity building, including the Basij motor courier *[Basij is a social institution with different functions to create the ability of people to help society when disasters and unexpected events occur. This institution plays a role in attracting, training, organizing, and employing public volunteers]*, financial support for women entrepreneurs, setting up mobile pharmacies, developing children's social skills (through campaigns, startups, online software, and competitions), and home workshops for fabric mask production. One of the participants said:

“*At the beginning of Corona, my business was closed … But I saw a clip on the Internet that a person was making money with a motorcycle in China. I decided to deliver my wife's homemade food to customers using a motorcycle. For this purpose, I designed a page on Instagram and started promoting my work. I even bought the essential items that people needed and could not go out due to the quarantine and delivered them to the customers … This work is still going on … I helped the health of the community and started my own business. This is the power of the media.”* (Participant 41)

Finally, one of the most important strategies used during the COVID-19 crisis was legal strategies, such as crisis management, guidelines, government compensatory policies, and prohibitions/restrictions (control of borders and social distancing). A member of Iran's National Headquarter Against COVID-19 said:

“*The government offered various strategies to limit the COVID-19, including ending trips, closing schools/universities, closing shopping mall, closing religious places, and banning religious meetings.” (Participant* 104)

## Discussion

### Overview

This study aimed to investigate social innovative strategies applied in Iran to increase CE in response to the COVID-19 crisis. The findings revealed several core concepts and strategies, including information giving/sharing, consultation, involvement/collaboration, health education/prevention, empowering, and advocacy. To identify the practical and innovative strategies that increase CE in a COVID-19 emergency, hypothetical basics were explored, and field documents were analyzed by recognizing themes, categories, sub-categories, and codes, which could help legislators in key strategic decisions as well as in creating strategies applicable for future pandemics. When faced with public health crises, such as COVID-19, countries should adopt approaches to increase the motivation for community participation in disease reduction (36). For instance, the schedules taken in China showed that quarantine and social distancing were able to stop the rapid spread of COVID-19 (34). The operationalization of health actions at the individual and social levels requires the full participation of the community, such as repeated handwashing, social isolation, and flexible job schedules (36).

### Theme 1: Information giving and sharing

We observed that correct, relevant, and up-to-date communication of health risks is a vital component of CE. In addition to improving community awareness and decreasing risky behaviors, this also contributed to promoting and maintaining trust. The findings highlighted that information giving/sharing regarding the COVID-19 crisis was done using public awareness campaigns, correct information management, new technologies and telecommunication tools, local advertising, and media infrastructure. Health professionals, government agencies, NGOs, and social media were trusted sources. An effective response in an epidemic is when accurate information about the burden of disease and death is told to society as soon as possible. This accurate dissemination of data allows the government and society to implement control and management methods quickly and on a large scale. Active communication and information sharing will similarly reduce the spread of “false information” and “infodemics” (37–39).

All three categories of participants believed that the dissemination of information was an effective approach in CE. The laypeople and healthcare providers believed that CE in crisis management could be increased when accurate statistics and information on deaths and the scope of the crisis were reported. Furthermore, the group of community leaders believed that while correct information should be published, it is not necessary to report all the details of the crisis to prevent fear, anxiety, and panic among the people. Key to fighting infodemics and advocating suitable communication will be recognizing and removing false news and gossips via the engagement of healthcare providers, community leaders, laypeople, and open channels for two-way communication between government authorities and community stakeholders. These leaders should be prepared to identify misinformation and to advocate correct, clear, and truthful information among communities, as well as to address and describe any modifications to the message.

Creating risk communication and disseminating information and crisis results to the community by health system reference groups can be effective. To successfully control the COVID-19 crisis or other health crises in future, comprehensive cooperation, strong government leadership, and multi-sectoral coordination are necessary to decrease misunderstandings and rumors and increase engagement with vulnerable people following capacity building, accurate communication, and resource mobilization. In this regard, digital health tools have been proposed as effective tools to support information sharing and communication, observation and monitoring, healthcare provision, and the expansion of vaccination (40). Digital health approaches can simplify the fast general sharing of data, encourage CE, and foster the participation and empowerment of people in implementation (41).

### Theme 2: Consultation

Consultation was another innovative strategy employed for raising CE during the COVID-19 crisis by getting feedback from the community, setting up public and open consultation systems, launching a self-assessment system, and implementing a comprehensive integrated system for psychological and social health services. Among the three categories of participants, healthcare providers played the most important role in providing advice to the community. Some of their counseling efforts included preventive measures, the promotion of vaccination, compliance with health protocols, and psychological counseling. The second group, which increased community participation by providing advice, was community leaders. They also invited society in different ways to cooperate with the health system and the government in the management of COVID-19. The third category (laypeople) received various counseling services, and most of their opinions were about how counseling and feedback were received.

Marsh et al. reported that consultation needs a reliable public image, where truth suggests a reasonable, stable, and precise demonstration of the several different populations in the community (42). Such demonstrations can be from governmental leaders, religious leaders, or respected people in the community. For instance, some studies specified that they consulted with and required the consent of native leaders of the community, already future participants of the community (43–45). In Tindana's study, it became clear that consulting with the trustworthy people of the community is not only a conventional necessity for obtaining consent from the community but also an opportunity to increase insights into cultural morals that may affect the topic (46, 47). This seems to be an exclusive story of CE in the multicultural setting of Iran, where community construction is determined and accepted and where there is some community cohesion (48). Similarly, Tedrow et al. recommended keeping consultations and discussions with leaders of the community during a crisis fairly simple (49).

### Theme 3: Involvement and collaboration

As Iran's COVID-19 epidemic grew, it became obvious that healthcare employees could not manage and control contact tracing or infection supervision. Community involvement is a manner of running straight with the community or via its agents to form a plan and perform the topic. The COVID-19 pandemic has helped as a modification originator in CE by using social innovative strategies in crisis management. The modifications were largely focused on the introduction of technology, with improvised and scheduled novel procedures for active CE.

A range of strategies for involvement or collaboration with the community applied and our work initially to and during the COVID-19 crisis has drawn on these, including networking, collaboration/lobbying between government and NGOs in crisis, multi-sectoral collaboration through COVID-19 committees, establishing platforms for community collaboration, social mobilization, shared leadership/decentralization/ability to control, managers' commitment, incentives/motivations, promoting a culture of participation, sensitizing, persuasion, moaning, and stakeholder engagement. According to the participants' views, decentralization of the decision-making process to the local level is effective in reducing resistance and increasing CE. This can be done directly or work indirectly by ensuring that the community has an impact on the target. Inviting the community to cooperate should be within the capacity of the community if the community wants to be motivated to engagement (17, 50).

All three categories of participants believed that involvement and collaboration were key components of COVID-19 crisis management. However, the role of community leaders was more important than that of the other two groups. By using different approaches, community leaders tried to encourage people to cooperate with the government and the health system in following health protocols and principles during the recent crisis. They provided the right context for community participation through encouraging, persuasion, motivational approaches, social mobilization, multi-sector collaborations, and sensitization. Healthcare providers were in the second category. The biggest role of this group was in persuading and sensitizing society. Finally, people ranked third in using this strategy. The biggest role of laypeople (according to their statements in this study) was in social mobilization and cooperation with NGOs. However, there was also a group of people who had created many problems for the government and the health system by violating quarantine rules.

### Theme 4: Health education and prevention

Most participants noted that health education and prevention strategies, including prevention, screening, quarantine, and maintenance, helped them to manage and fight against the COVID-19 pandemic, as well as engage the community. We believe these to be respectable suggestions that emphasize the fact that communities are becoming more aware of elements that endanger their lives. The use of web-based educational social media has been identified to help consider health emergencies and provide access to suitable evidence of troubling events (51). Public interventions may finally take on several practices. Given that the content is general information on restricted interactions by means of appropriate handwashing methods, screening passengers, and approving good quarantine approaches, these interventions can also be of vast advantage to the community (52).

All three groups of participants played an important role in providing health education to the community to manage the COVID-19 pandemic. The roles of each of the three study groups varied depending on the location and conditions. For example, laypeople participated more in complying with the principles and instructions, complying with quarantine, and complying with the principles of risk communication. The healthcare providers contributed to attracting CE through various face-to-face and virtual training, screening, and vaccination. The community leaders also took an important step in increasing CE to control this crisis by establishing laws in offices and public places about mask-wearing and social distancing, national coordination, establishing quarantine, and providing a suitable platform for screening.

### Theme 5: Empowerment

According to findings, jihadi/voluntary/faith-driven actions, social trust building, social responsibility, community resiliency, and strengthening society's efficiency/finding talent were some of the strategies applied to empower CE during the COVID-19 crisis in Iran. Community empowerment is the procedure that advances their properties and powers and creates the ability to reach access, allies, and networks to achieve management. Shared leadership was perceived to be of significance in increasing CE. Community empowerment should be planned, made, and directed in the community if it was to be operative and stable in decreasing the community's risk for COVID-19 and raising and maintaining their wellbeing and rights (53). The occurrence of the COVID-19 crisis as a pandemic has a socioeconomic and mental influence on the community. Consequently, to support the community in facing challenging periods due to the COVID-19 crisis is a public responsibility. Strategic policymaking is required to create a helpful, safe, and easy atmosphere for the community. Community empowerment considers the financial, sociocultural, and political elements that strengthen health, and creates cooperation with other regions in discovering answers. Health administrators and communities co-recognize complications and implement solutions by empowering community constructions or local organizations to provide variation (54).

The findings on this theme showed that the laypeople had a high level of compliance with the community leaders' and healthcare providers' actions on the management of the COVID-19 crisis at all levels, including readiness to be empowered by voluntary and faith-driven actions; building social trust; building social responsibility; building community resiliency; strengthening society efficiency; and finding talent. We found that community groups, religion groups, and key stakeholders (laypeople—youth, women, and the elderly) also participated in empowering and building trust. Trust and confidence are important elements of CE, and if communities lack confidence, they will likely abstain from their healthcare providers and guidance from government officials and community leaders (47, 55). We observed that risk communication, which is specified by interacting with native communities, deeply affected the desire to prepare for crises and the specified grade of community confidence and trust in, and finally compliance with, the government's preventive actions. Our findings also indicate that creating and supporting community cooperations, which are based on operative collaborations and constant communication, can be significant in creating and maintaining trust, finally helping CE. Consequently, reinforcing association and trust is recommended to ensure the stability of community health promotion programs in future.

### Theme 6: Advocacy

Another important strategy for facilitating CE is advocacy through capacity building, coalition, leaders' involvement, mobilizing community groups, and legal and policymaking strategies. These were observed to be useful in guiding management, prevention, and control strategies and enabling the combination of these measures in the usual work of all groups. Operative advocacy is a vital means in the attempts to assist the most susceptible persons throughout the COVID-19 crisis. Although the COVID-19 crisis may have transformed how advocacy is implemented, collaborating with selected administrators and strategic decision-makers during this period of the COVID-19 pandemic was vital to obtaining funds and other support. The community background and the functioning of the elements can generally impact the success of CE, with innovative strategies being addressed in the broader structure of their implementation (56, 57). This may include promoting and building community capacity and advocative and supportive settings for engagement, advocating connections, and advocative policy and funding settings, as well as creating environments of respect, confidence, and shared subcultures, norms, and targets (17, 25, 58).

The participation of local leaders (laypeople) who had high levels of respect was very important in advocating for healthcare providers and government/community leaders in managing the COVID-19 crisis. Therefore, without their advocacy, participation, and cooperation, COVID-19 management measures would not be implemented properly. However, the role of the government and healthcare providers is not hidden from citizens. Notably, laypeople had an effective role in mobilizing local resources and volunteers and using social media devices such as Instagram and WhatsApp to gather and send preventing COVID-19 messages. However, these measures would not have been very successful without the advocacy of government agencies and community leaders.

The key concepts of the full community strategies are considerate community difficulty, identifying community abilities and necessities, raising connections with communities, constructing and keeping cooperations, empowering native acts, and persuading and reinforcing social substructures, links, and resources (59). Public strategies for CE include community involvement, mobilization, cooperation, and empowerment (3, 60), usually during the planning and implementation of interventions (17, 24). Particular applications of CE include awareness, increasing sensitization, consultation, building capacity via teaching and management, growing public consistency (constructing networking and reliance), and reinforcing relationships with health organizations. Regarding COVID-19 in Iran, the key CE actions included operative communication for socio-behavioral variation, observation, and contact tracing (17).

### Strengths and limitations

In the current study, social innovative strategies for increasing CE were recognized using a qualitative method. Other innovative strategies for increasing CE in crisis management that has not been mentioned or reported in this qualitative study may be relevant. In future studies, mixed-method approaches can be applied. We acknowledge that the strategies identified and applied to increase CE based on the perspective of the study sample provided insights regarding the crisis across Iran. Although some strategies for increasing CE identified are related to multiple cultures and contexts, some may be culture dependent. There are different countries where there is a lot of study with a particular sociocultural, political, economic, and environmental perspective. Thus, those results may not be applicable and appropriate to other cultures, and the results may not be fully generalizable.

This relatively average-scale study aimed to discover social innovation strategies for increasing CE in combating COVID-19, focusing specifically on the multicultural society of Iran. The research provinces were purposively selected for practical and source reasons. We surmise that this sample delivered general viewpoints regarding the crisis across Iran. However, due to the remarkable diversity of pandemics and health crises, our findings may not be completely generalizable. More research is needed to investigate the impact of COVID-19 on large-scale communities as well as other vulnerable groups. Nevertheless, we collected and triangulated information from three groups of stakeholders and actors to combine themes generated from more than one source, thus controlling for the effect of bias.

### Implications and recommendations for future studies

Our study underlines hopeful findings in the use of CE innovative strategies, recommending that for crisis management and control of pandemics, CE may be significant and influential in engaging with the public. Further study is needed to explore whether social innovative strategies of CE may be effective in the control and management of other kinds of health crises in all groups and cultures. Future publications and articles on the synergies of CE via social innovative strategies in health crisis management and control should place more emphasis on community involvement, advocacy, health promotion strategies, community consultation, community mobilization, and community empowerment. There is also a need to conduct further studies focusing on infectious diseases in this field. Even though some groups of communities (three groups of community stakeholders with different cultural characteristics) were interviewed, there are many more that may not have been studied, and their insights and experiences should be taken into account in future research.

## Conclusion

The spread of the COVID-19 pandemic required social and community-based responses. These reactions increased the possibility of fair access to health services, especially among vulnerable groups and minorities. As with other epidemics, applying the experience of the comprehensive participation of communities played an important and active role in the management, prevention, and control of the COVID-19 crisis. In this regard, giving and sharing information, community consulting, involvement and collaboration, health education/prevention approaches, community empowerment, and advocacy were the most important innovative strategies encouraging the community to perform COVID-19 crisis management and control. The findings of the present study offer the basis for advancing social innovation strategies for raising CE based on associations between government, NGOs, and the community, leading to sustainable progress in societies. However, the effort to build and strengthen community capacity must be placed within the scientific efforts made during the COVID-19 crisis.

## Data availability statement

The original contributions presented in the study are included in the article/Supplementary material, further inquiries can be directed to the corresponding authors.

## Author contributions

MK-P designed the project, collected the data, analyzed the data, and wrote the first draft of the manuscript. TP participated in analyzing the data. MK-P and KP critically revised the final article. All authors read and approved the final manuscript.
