# Supplementary material for: Social innovation in health and community-driven engagement as a key strategy for addressing COVID-19 crisis challenges: insights and reflections from the multicultural society of Iran
Source: Front Public Health. 2023 Jun 6;11:1174385. doi: 10.3389/fpubh.2023.1174385 (PMC10279867; doi:10.3389/fpubh.2023.1174385)
Supplement: Supplementary file 1 [file Data_Sheet_1.DOCX]

***Appendix* 1: Conducting Individual In-depth Interviews guideline:**

1. **Planning the study**

The researchers planned the study with the input of the stakeholders, and research experts to determine:

• **What they want to learn**

**• How to apply what they learn**

**• Their budget**

**• Their criteria for the participants. They selected people who were:**

- Key informants hold special and expert knowledge about the phenomenon to be studied, and are willing to share information and insights. They helped the researchers to validate their ideas and perceptions with those of the key informants.

- The researchers looked for participants who had shared an experience, but varied in characteristics and in their individual experiences. For example, a qualitative study on the experiences will recruit participants varying in age, language, culture, and job and educational level.

- The number of interviews or groups they conducted

- The main data collection method that used in the study was in-depth interviews; the researchers conducted about 187 interviews. The researchers continue sampling only until data saturation had been achieved. Data saturation means the collection of qualitative data to the point where a sense of closure is attained because new data yield redundant information.

- **Who conducted the research**

In total, seven trained interviewers, who were familiar to the culture of the selected provinces as well as the qualitative research method, conducted semi-structured 40-minute telephone interviews with 187 participants. Considering the dispersion of the study setting, the cultural and linguistic diversity of the research community and the high prevalence of COVID-19 in the time efficiency of the study, four researchers familiar with the qualitative research method were invited for administrative coordination to obtain participants' contact numbers. In this regard, for the provinces of Khuzestan, Zanjan, Lorestan, and Golestan, which had various languages and dialects, native researchers who were also familiar with qualitative research methods were invited to study. The first author of the article (MK) conducted interviews in Mazandaran and Tehran provinces, and the second author of the article (TP) conducted interviews in Kurdistan province due to familiarity with the local language of that region. The reason for this choice was that these people were familiar with the culture, language and religious characteristics of the mentioned areas. After the administrative coordination with different departments by each of the researchers in the selected provinces, the contact numbers of the individuals were received to obtain permission and coordinate the time of the interview.

1. **Choose the location and format for interviews**

**Semi-structured in-depth Interviews (IDIs)**

The interviews were conducted over android smartphones that had the call-recording property. Telephone interviews were applied due to the physical distancing protocol and the necessity to maintain safety while conducting the qualitative study. Each interview continued for not above 40 minutes to evade affecting weariness to interviewees. Most of the interviews were held in Farsi (the official language of Iran). In cases where the participant preferred to speak his native (mother tongue) language or could not speak Farsi fluently, the interview was held in the native language of the same region (that was the reason for choosing interviewers familiar with the language of the selected provinces). Interviews were recorded and transcribed verbatim in Farsi. Considering that some of the interviews were conducted in the native language of the people of that area, at the end of that day, they were implemented in Persian by the same interviewer. The transcripts and digital recordings were cross-checked.

1. **Screen recruitment**

The participants who refused to have an interview because of their tight schedule and those who were reluctant to participate in the interviews were removed from the study.

1. **Recruit participants**

We tried to choose the right participants for the interviews. Informed consent was obtained from each participant who participated in the study. Then, the purpose of the study was described for the participants and ensured participants about the anonymity and confidentiality of the information. Then, the interviews were started based on three main open-ended and non-directive questions, which were followed by appropriate probe questions. Assuredly, the participants were free to leave the study whenever they wanted (i.e., feeling uncomfortable or tired).

1. **Develop a moderator’s guide**

The quality of the moderating guide is critical to your success. The moderator's guide told the interviewer what information they want from the participants and helped her keep the discussion on track and on time. This guideline was consisted of the aim of the study and main interview questions.

The study aim was to identify social innovative approaches that might increase community participation in response to the COVID-19 crisis. The interviews were focused on the three main following questions:

How was your experience about engagement in management and control of COVID-19 pandemic?

What strategy and procedure you have applied for engagement in management COVID-19 pandemic?

How strategy and procedure affect your engagement in this regard?

Based on responses to the questions, follow-up questions were asked, such as, “Tell me all about your role in COVID-19 crisis management and control?”, “How were you wedged by the COVID-19 crisis?” “What is your opinion about the role of CE in COVID-19 management and control?”, “How did you perform this role?”, “What obstacles did you face?”, “To what extent did the government receive the contribution of the population? How was this influenced?”, “What challenges did you encounter in the process of COVID-19 prevention in the areas? What strategies did you use in solving the challenges?”, and “What can be done further?” After each question, participants were invited to explain more about what they had reported. For example, they were asked, “What do you mean?” or “Explain more” for a deeper consideration of the participant’s experiences regarding the topic. In fact, the authors asked open-ended questions, so participants could provide more in-depth responses than just “yes” or “no.” Also, they attempted to use the questions that weren't worded in a way that would prompt a particular response. This helped to ensure that participants offered honest responses, not the answers they think the authors wanted.

1. Conduct the interviews

The in-depth interviews began with the interviewer welcoming participants and explaining to them the process (e.g., that there are no right or wrong answers, the considerations about they confidentially would be taken into account, that the session would be recorded, the participants were free to leave the study whenever they wanted (e.g., feeling uncomfortable or tired)), and the interviewer explained to them the aim of the study and requested them to ask any questions if they wanted.

***Appendix 2:*** Interview guides for key experts and policymakers regarding Social Innovation in Health and Community-Driven Engagement as a Key Opportunity to Address COVID-19 Crisis Challenges

Thank you for agreeing to participate

The interview will take maximum 40 minutes. I would like to ask your permission for taping the session because I do not want to miss any of your ideas and suggestions. Although I am going to take some notes during the session, I cannot possibly write fast enough to get it all down. Because we are on tape, please be sure to speak up so that we do not miss your ideas. I want to note again that the information you provided will be confidential. Any information we use from your interview will be combined with information from other participants.

Do you have any questions before we start the interview?

**Participant profile**

| Age: |
| --- |
| Gender: |
| Marital status: |
| level of education: |
| Occupation: |
| Nationality: |
| Religion: |
| Province of residence: |
| Language / Accent: |
| Experience of Social Innovation in Health: |

Before we start our discussion, can you tell me about yourself?

Educational background, service year, working department?

How was your experience about engagement in management and control of COVID-19 pandemic?

What is your definition of social innovation in health, and how they are formed?

How do you think social innovation changes behaviors, processes and policies during the crisis?

What is your opinion on the role of community participation in the management of the COVID-19 crisis?

How do you think social innovation leads to community participation in crisis management?

What strategy and procedure you have applied for engagement in management COVID-19 pandemic?

How strategy and procedure affect your engagement in this regard?

What were the challenges you faced to increase community participation in controlling the COVID-19 crisis in your city?

Do you have any questions for us or something you want to add?

Thank you very much for your time

***Appendix 3:*** Interview guides for Iranian citizens regarding Social Innovation in Health and Community-Driven Engagement as a Key Opportunity to Address COVID-19 Crisis Challenges

Thank you for agreeing to participate

The interview will take at least 40 minutes. I would like to ask your permission for taping the session because I do not want to miss any of your ideas and suggestions. Although I am going to take some notes during the session, I cannot possibly write fast enough to get it all down. Because we are on tape, please be sure to speak up so that we do not miss your ideas. I want to note again that the information you provided will be confidential. Any information we use from your interview will be combined with information from other participants.

Do you have any questions before we start the interview?

**Participant profile**

| Age: |
| --- |
| Gender: |
| Marital status: |
| level of education: |
| Occupation: |
| Nationality: |
| Religion: |
| Province of residence: |
| Language / Accent: |
| Experience of Social Innovation in Health: |

Tell me about your experiences during the COVID-19 pandemic in Iran?

How was your experience about engagement in management and control of COVID-19 pandemic?

What are the effects of the COVID-19 pandemic on your health, interfamily relationships, and work?

What are the most serious challenges and problems of you and your family during the COVID-19 pandemic in your city/village?

What is your opinion about the role of community participation in the control and management of the Covid-19 crisis and how it might have an impact?

What innovative and people-friendly methods and strategies do you suggest to increase community participation in the management of the COVID-19 crisis in your city?

How do you think social innovation leads to community participation in crisis management, especially during the COVID-19 epidemic?

What strategy and procedure you have applied for engagement in management COVID-19 pandemic?

How strategy and procedure affect your engagement in this regard?

Thanks for your time and information, have a good day!!!
